# A mixed methods study on poisoning and injury-related emergency department visits associated with opioids in Canada, 2011 to 2022: from the Canadian hospitals injury reporting and prevention program

**DOI:** 10.1186/s12889-024-20016-8

**Published:** 2024-09-18

**Authors:** Xiaoquan Yao, Alyssa-Ann Rama, Julianna Mazzitelli, Steven R. McFaull, Wendy Thompson

**Affiliations:** The Public Health Agency of Canada, 785 Carling Avenue, Ottawa, ON K1S 5H4 Canada

**Keywords:** Emergency room visits, Wounds and injuries, Poisoning, Opioid, Naloxone, Bystander, Social resource, Sentinel surveillance, The Canadian Hospitals Injury Reporting and Prevention Program, Mixed methods research

## Abstract

**Background:**

The opioid crisis is a serious public health issue in Canada. There have been many surveillance programs and research studies on opioid-related emergency department (ED) visits at a national, provincial, regional or municipal level. However, no published studies have investigated the in-depth contexts surrounding opioid-related ED visits. In addition, few studies have examined injuries other than poisonings in those visits. The objective of this study is to investigate the contextual factors and co-occurrence of poisonings and injuries among the opioid-related ED visits in a Canadian sentinel surveillance system on injuries and poisonings from 2011 to 2022.

**Methods:**

This study used a mixed methods design. The data source was the Canadian Hospitals Injury Reporting and Prevention Program. We first selected all opioid-related ED visits during our study period and then identified the contextual factors through a content analysis of the combination of the narrative description and other variables in the patients’ records. The contextual factors were organized into themes as opioid use context, social resource utilization, bystander involvement, and prior naloxone use. The opioid use context was used as a co-variable to examine the other themes and ED presentations (poisonings and other injuries). Quantitative descriptive approach was used to analyze all the contexts and ED presentations.

**Results:**

The most common opioid use context was non-prescribed opioid use without intention to cause harm, followed by self-poisoning, children’s exposure, and medication error. Various rare contexts occurred. Paramedics participated in 27.9% of visits. Police and security guards were involved in 5.1% and 2.3% of visits, respectively. Child welfare or social workers were involved in 0.4% of visits. Bystanders initiated 18.9% of the ED visits. Naloxone use before arriving at the ED occurred in 23.4% of the visits with a variety of administrators. The majority of patients presented with poisoning effects, either with poisoning effects only or with other injuries or conditions.

**Conclusions:**

Our study has provided an in-depth analysis of contextual factors and co-occurrence of poisonings and injuries among opioid-related ED visits in Canada. This information is important for ED programming and opioid-related poisoning and injury intervention and prevention.

**Supplementary Information:**

The online version contains supplementary material available at 10.1186/s12889-024-20016-8.

## Background

The opioid crisis is a serious public health issue in Canada. There were 42,494 apparent opioid toxicity deaths between January 2016 and September 2023 [1], with unregulated opioid, fentanyl, contributing to the majority of the deaths [1, 2]. The COVID-19 pandemic exacerbated the crisis. Between 2019 and 2021, the number of opioid-related deaths increased 107% and the annual years of life lost doubled in nine Canadian provinces and territories [3]. More recently, approximately 22 apparent opioid toxicity deaths occurred each day between January and September 2023 [1]. The opioid-related poisoning hospitalizations and emergency department (ED) visits increased in 2023 compared to the same period in 2022 [1]. This situation highlights the continued need for surveillance and research to inform public health strategies to curb this crisis.

People who consume opioids may suffer from the toxicity (also referred to as poisoning or overdose). They may also sustain other injuries related to opioid use, such as unintentional falls, self-inflicted cuts, etc. In this paper, we use injuries to refer to any injuries except poisonings. EDs are key access points to the health care system for those suffering from opioid-related poisonings and injuries. ED visit data can provide important evidence for policy makers. There have been many surveillance programs and research studies on opioid-related ED visits at a national, provincial, regional or municipal level. These studies have highlighted the magnitude of opioid-related ED visits (counts and rates) and their time trends [4–15]. Some, to a limited extent, examined the demographic characteristics [5, 10, 13, 15] or risk factors [6] associated with the ED visits. To our knowledge, no published studies have investigated the in-depth contexts surrounding poisoning and injury-related ED visits associated with opioids in Canada, such as the opioid use context, social resource involvement, or whether naloxone was used before visits, etc. In addition, most studies focused on poisonings; few studies have examined injuries among those visits. This level of information would provide a better understanding of those ED visits, such as the comorbidities of patients, the factors contributing to the poisonings and injuries, other resources associated with ED visits and prior naloxone treatment. EDs are critical places for opioid-related poisoning and injury intervention and prevention [16–19]. Therefore, analyzing poisoning and injury-related ED visits associated with opioids could provide important information for ED programming, intervention and prevention. The objective of this study is to investigate the contextual factors and co-occurrence of poisonings and injuries among the opioid-related ED visits in a Canadian sentinel surveillance system on injuries and poisonings from 2011 to 2022.

## Methods

### Data source

We used data from the Canadian Hospitals Injury Reporting and Prevention Program (CHIRPP), an injury and poisoning sentinel surveillance system funded and administered by the Public Health Agency of Canada [20]. CHIRPP collects ED visit data, currently operating in 11 pediatric and nine general hospitals across Canada [21]. At the time of an ED visit, a patient or their companion (e.g. parents/guardian) provides the details of injury and poisoning events leading to the visit, such as time, place, person involved, substance, contributing factors and history etc. CHIRPP recorded the information using variables including a free-text narrative that allows an in-depth description of the events. CHIRPP also collects clinical details including substance test results, diagnosis (e.g. nature of injuries and poisonings, body parts affected), and treatment received. Details about the development, use, and data quality control of CHIRPP have been published elsewhere [20]. Each CHIRPP-participating hospital has undertaken their own ethics review. No additional ethics review was required for this study. Data in CHIRPP have been de-identified.

### Study sample selection

We retrieved records from the CHIRPP database on January 12, 2023 and re-visited it on August 1, 2023. We first chose all the records with an injury or poisoning date between April 1, 2011 and December 31, 2022 and then identified opioid-related ED visits. In the identification process, we first used SAS Perl regular expression [22] to search through the text variables in CHIRPP (i.e. narrative and substance). The search terms included opioids and its related products, i.e. prescription medication, over-the-counter medication containing opioids as well as illicit drugs. The terms included general names, brand names, selected street drug names, and their corresponding French terms. We also considered synonyms, truncated terms, different spellings and possible misspellings to improve search sensitivity (see Appendix 1 for the comprehensive list of search terms).

**Fig. 1:**
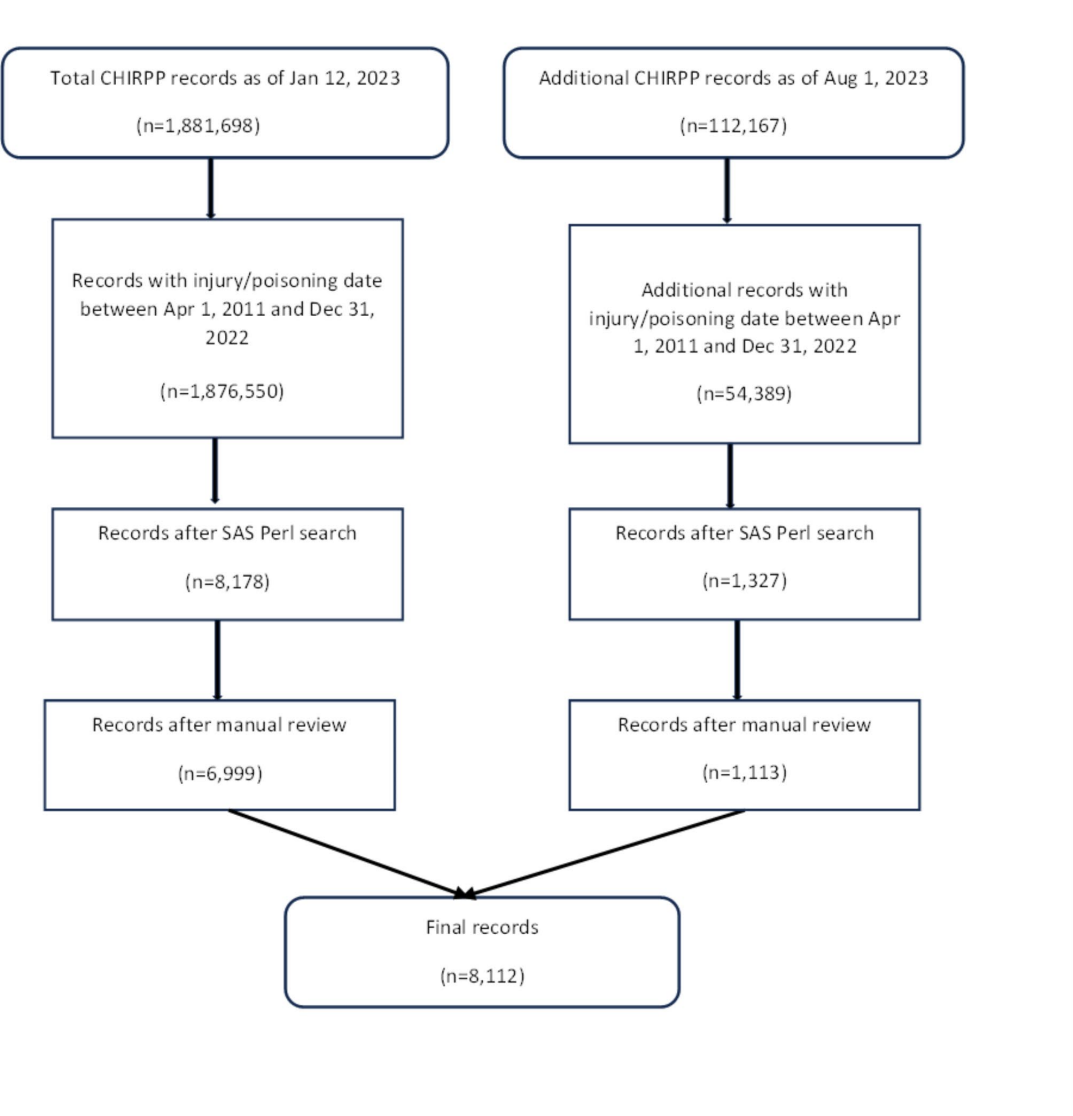
Study sample selection process

After the initial search, we reviewed all identified records and excluded ones where the poisoning or injury event was not related to opioids (e.g. taking opioid pain medication after the reported injury event for pain relief). There were instances where the eligibility of records was unclear. We applied the following inclusion/exclusion criteria to these records. First, if a record reported only historic opioid use and the use was clearly related to the current ED visit, the record was included; otherwise, the record was excluded. Second, if naloxone was used to treat a suspected overdose and was effective (clearly indicated), the record was included despite no opioids specified. However, if the narrative indicated that naloxone did not have any effect and no opioids were specified, the record was excluded. In total, 8,112 opioid-related ED visit records were included in this study. Figure 1 illustrates the study sample selection process.

### Qualitative content analysis

We used a mixed methods design, a qualitative content analysis and then a quantitative descriptive analysis. First, we identified the contexts through a content analysis of the combination of narrative, age, sex, diagnosis, and time/place of poisoning and/or injury events. We adopted a combined deductive and inductive approach for the content analysis [23, 24]. We created an initial coding framework based on a previous analysis [6] on contextual factors surrounding suspected opioid-related visits. The principal investigator (XY) read and re-read the records to identify categories and sub-categories of opioid use context and performed coding based on the framework, provided a new code to any new category or sub-category, and added or collapsed codes based on the ongoing interpretation of data. This process was iterated for more than 3000 records. Then, a coding table was established, in which an operational definition for each category/sub-category was written. AR and JM tested the operability of the coding table using 200 randomly chosen records. After that, AR and JM coded all records from 2011 to 2022. During the process, any new emerging category/sub-category continued to be incorporated into the coding table. The research team held weekly meetings to discuss coding issues. For example, when the narrative was ambiguous, we discussed whether we should follow up with the CHIRPP sites to get a clarification or we could interpret the narrative under reasonable assumptions based on other information in the record or we should code it as unclear. During the process, social resource related information, such as paramedics, police, security guards, child welfare or social workers, bystander involvement, and prior naloxone use (including types of administrators) emerged from the narratives, so we performed coding on the above information. To check the inter-rater reliability [25], AR and JM both coded a subset (*n* = 1,327) whose records were entered into CHIRPP between January 12 and August 1, 2023 and selected after the SAS Perl search (see Appendix 2 for the test results).

### Quantitative descriptive analysis

After the categories and sub-categories of opioid use context were identified, we used a quantitative descriptive approach to examine the distribution of the categories of opioid use context and the distribution of age, sex, opioid, and concomitant substance use for each category of opioid use context. For the other contextual themes (social resource utilization, bystander involvement, and prior naloxone use) and ED presentations (poisonings and injuries), opioid use context was used as a co-variable to describe the distribution. We grouped ages into < 1, 1–4, 5–11, 12–17, 18–24, 25–44, 45–64, and 65 and over (in years). Sex in CHIRPP is the sex at birth. Since CHIRPP has not started to collect gender information, we were not able to perform gender-specific analysis.

We used SAS 9.4 [26] to do statistical analyses. By assuming that a theoretical opioid-related CHIRPP super population existed and our sample were drawn from the infinite samples, we constructed 95% confidence intervals (CI) for proportions (Wald intervals for 25–75% and logit intervals for < 25% or > 75%) [27]. Chi-square test was used to examine the statistical significance of differences in proportions. In any situation where chi-square test could not be applied due to small cells, some categories were pooled to perform the test. After pooling, if small cells were still an issue, an exact test was used. *P* value was set at 0.05.

## Results

### Sample characteristics

Table 1 presents the age, sex, and temporal distributions of opioid-related visits, compared with all visits in CHIRPP. More males than females were observed among both opioid-related and all CHIRPP visits. Patients among opioid-related visits tended to be older than those among all CHIRPP visits. The proportions of opioid-related visits among all CHIRPP visits were much higher during 2018–2022 than in the previous years. Of note, we expect the possibility of data processing delay for 2022 is much greater than the previous years, so we cannot conclude 2022 had less visits than 2021.

**Table 1:** Characteristics of opioid-related records compared to all CHIRPP records, 2011–2022

	Opioid-related records	All CHIRPP records	Incidence of opioid-related records per 1,000 CHIRPP records
	*n*(%)	*n*(%)	
**All**	8,112 (100%)	1,930,939 (100%)	4.2
**Sex** ^#^			
Male	5,216 (64.3%)	1,074,635 (55.7%)	4.9
Female	2,891 (35.6%)	855,960 (44.3%)	3.4
Not specified	5 (0.1%)	344 (0.0%)	14.5
Median age in years[IQR]*	33.3 [19.3]	11.8 [12.9]	Not applicable
**Age group (years)** ^#^			
< 1	52 (0.6%)	73,339 (3.8%)	0.7
1–4	265 (3.3%)	408,186 (21.1%)	0.6
5–11	34 (0.4%)	496,379 (25.7%)	0.1
12–17	1,134 (14.0%)	474,259 (24.6%)	2.4
18–24	671 (8.3%)	83,160 (4.3%)	8.1
25–44	4,059 (50.0%)	161,839 (8.4%)	25.1
45–64	1547 (19.1%)	126,359 (6.5%)	12.2
65+	203 (2.5%)	106,601 (5.5%)	1.9
Unknown	147 (1.8%)	817 (0.0%)	179.9
**Injury period** ^#^			
2011	63 (0.8%)	95,014 (4.9%)	0.7
2012	64 (0.8%)	132,620 (6.9%)	0.5
2013	83 (1.0%)	141,248 (7.3%)	0.6
2014	99 (1.2%)	144,161 (7.5%)	0.7
2015	111 (1.4%)	144,410 (7.5%)	0.8
2016	113 (1.4%)	151,314 (7.8%)	0.7
2017	323 (4.0%)	167,028 (8.7%)	1.9
2018	807 (9.9%)	189,464 (9.8%)	4.3
2019	1,046 (12.9%)	195,815 (10.1%)	5.3
2020	1,251 (15.4%)	176,784 (9.2%)	7.1
2021	2,642 (32.6%)	219,059 (11.3%)	12.1
2022	1,510 (18.6%)	174,022 (9.0%)	8.7

Note: (1) ^#^*P* < 0.05 for the distribution difference between opioid-related and all CHIRPP records. (2) ^*^Excludes records with unknown age (n_opioid-related_=147, n_all CHIRPP_=817)

### Opioid use context

We classified opioid use context into ten main categories which include their sub-categories (Table 2). The terms “medication” and “drug” are often used interchangeably; however, in this paper, we used “medication” to refer to an opioid that was manufactured by a pharmaceutical company, approved for medical purposes in humans, and prescribed by a doctor to a patient. “Drug” was used to refer to illegal, unregulated opioids or pharmaceutical opioids not prescribed to a patient or used differently than prescribed [1]. 

Table 2 presents the categories and sub-categories of opioid use context and its characteristics, i.e. the distribution of age, sex, opioid type (some patients were related to multiple opioid types) and concomitant substance use. We can see that the categories were associated with different age and sex profiles; there was also discrepancy in the use of opioids and other substances. The most common category was non-prescribed opioid use without intention to cause harm (50.8%, 95% CI: 49.7 – 51.9%), which included an opioid was used due to dependence, for euphoric effect (such as recreation use) or stress relief, an opioid *drug* was used for manage health conditions, or rehabilitation-related methadone/suboxone was concomitantly used with other *drugs*. Among this category, the median age was 33.6 years and males accounted for 68.9%. The five most common occurring opioids were fentanyl, heroin, oxycodone, codeine and morphine. 34% of cases involved other substances than opioids. The second most common category was self-poisoning (11.5%, 95% CI: 10.8 – 12.2%), with the median age of 18.1 years and 38.7% being males. The five most often occurring opioids were codeine, oxycodone, hydromorphone, morphine and fentanyl. 73% of cases involved other substances. Other less common categories included children’s exposure (3.6%), medication error (2.7%), medical treatment (0.7%), poisoned by others (0.2%), and mother used opioids during pregnancy or breast-feeding period (0.1%).

We grouped some rare contexts, such as drug concealing in organs (ex. gastrointestinal tract), second hand exposure on job duty, or taking opioids due to instructions from auditory hallucinations caused by mental illnesses. For some records, available information was not enough for us to determine the clear context; we were certain that some patients had no intention to cause harm to self (21.2%) while could not determine the intention for others (8.9%).

**Table 2:** Opioid use context and associated characteristics

Category ID	Category	Sub-Categories	Count (%) [95% CI]	% Male	Median age in years [IQR]	Five most common opioids (%)^*^	% Involving substances other than opioids
I	Non-prescribed opioid use, without intention to cause harm	1. An opioid was used due to dependence (opioid use disorder), for euphoric effect (such as recreational use) or stress relief. The opioid can be a drug or medication not used for the prescribed purpose. 2. Opioid ***drugs*** (not prescribed medication) were used to manage pain, insomnia, withdrawal symptoms from other substances, or other health conditions. 3. Rehabilitation-related methadone/suboxone was used concomitantly with other ***drugs***.	4,120 (50.8%) [49.7 – 51.9%]	68.9%	33.6 [15.9]	1. 53.8% - Fentanyl 2. 22.6% - Heroin 3. 3.2% - Oxycodone 4. 1.7% - Codeine 5. 1.7% - Morphine	34.3%
II	Medication error	1. A prescribed opioid medication was used and doctors’ instructions were unintentionally broken by self or caregiver, such as using wrong medication, expired medication, wrong dosage, interval, or administration route. 2. Opioid **medication** was **occasionally** used to treat pain without doctors’ instructions.	215 (2.7%) [2.3 – 3.0%]	50.7%	51.6 [45.7]	1. 24.2% - Hydromorphone 2. 23.3% - Morphine 3. 18.1% - Oxycodone 4. 14.0% - Codeine 5. 11.2% - Methadone	39.1%
III	Children’s exposure	1. Children ingested or absorbed adults’ opioid medication or drug. 2. Children were suspected of ingesting or absorbing adults’ opioid medication or drug.	295 (3.6%) [3.3 – 4.1%]	53.6%	2.1 [1.4]	1. 23.1% - Oxycodone 2. 20.0% - Codeine 3. 13.9% - Hydromorphone 4. 11.9% - Buprenorphine 5. 8.1% - Morphine	38.0%
IV	Mother’s opioid use	Mother used opioids (medication or drugs) during pregnancy or breast-feeding period.	10 (0.1%) [0.1 – 0.2%]	60%	0.1 [0.2]	1. 50.0% - Methadone 2. 20.0% - Codeine 3. 10.0% - Oxycodone 4. 10.0% - Fentanyl 5. 10.0% - Hydromorphone	40.0%
V	Medical treatment	1. Opioid medication was prescribed for managing pain. 2. Opioid medication was prescribed for rehabilitation.	53 (0.7%) [0.5 – 0.9%]	66%	41.6 [24.5]	1. 22.6% - Hydromorphone 2. 20.8% - Methadone 3. 13.2% - Buprenorphine 4. 13.2% - Morphine 5. 11.3% - Oxycodone	20.8%
VI	Other context, without intention to cause harm	1. Drug concealing in organs (ex. gastrointestinal tract). 2. Second hand exposure on job duty. 3. Taking opioids due to instructions from auditory hallucinations caused by mental illnesses.	32 (0.4%) [0.3 – 0.6%]	62.5%	28.4 [12.0]	1. 31.3% - Heroin 2. 21.9% - Fentanyl 3. 18.8% - Morphine 4. 9.4% - Methadone 5. 9.4% - Hydromorphone	28.1%
VII	Unclear context, without intention to cause harm	1. It was hard to differentiate between opioid misuse, medication error and medical treatment. 2. The context was not clear, but it was sure that there was no intention to cause harm.	1,720 (21.2%) [20.3 – 22.1%]	67.9%	35.3 [15.7]	1. 38.5% - Fentanyl 2. 16.8% - Heroin 3. 3.6% - Oxycodone 4. 3.1% - Methadone 5. 2.0% - Codeine	23.0%
VIII	Self-poisoning	1. Suicide attempt. 2. Self-poisoning without suicidal intent, such as grabbing attention.	930 (11.5%) [10.8 – 12.2%]	38.7%	18.1 [23.3]	1. 29.3% - Codeine 2. 18.0% - Oxycodone 3. 13.0% - Hydromorphone 4. 11.9% - Morphine 5. 10.8% - Fentanyl	73.0%
IX	Poisoned by others	Opioids were used by other people to deliberately cause harm to a patient.	18 (0.2%) [0.1 – 0.4%]	11.1%	17.4 [13.0]	1. 66.7% - Fentanyl 2. 11.1% - Heroin 3. 5.6% - Methadone 4. all the others - unspecified opioids	38.9%
X	Unclear context, undetermined intent to cause harm	1. It was hard to differentiate between unintended poisoning, self-poisoning and poisoned by others. 2. The context was not clear and the intention was not clear.	719 (8.9%) [8.3 – 9.5%]	72.8%	37.1 [16.6]	1. 53.9% - Fentanyl 2. 7.4% - Heroin 3. 3.1% - Oxycodone 4. 2.8% - Hydromorphone 5. 2.5% - Methadone	19.7%
Total			8,112 (100.0%)				

Note: (1) ^*^ Some patients were associated with multiple opioids. (2) The distributions of sex, age group, opioid type, and other substance were tested between Category I (reference) and each of the other categories:

sex — *P* < 0.05 for all pairs except for (I, IV), (I, V), (I, VI), and (I, VII);

age group — *P* < 0.05 for all pairs except for (I, VI);

fentanyl — *P* < 0.05 for all pairs except for (I, IX) and (I, X);

heroin — *P* < 0.05 for all pairs except for (I, IV), (I, VI), and (I, IX);

oxycodone — *P* < 0.05 for all pairs except for (I, IV), (I, VI), (I, VII), (I, IX) and (I, X);

codeine — *P* < 0.05 for all pairs except for (I, V), (I, VI), (I, VII), (I, IX) and (I, X);

morphine — *P* < 0.05 for all pairs except for (I, IV), (I, VII), (I, IX) and (I, X);

hydromorphone — *P* < 0.05 for all pairs except for (I, IV), (I, VII), (I, IX) and (I, X);

methadone — *P* < 0.05 for all pairs except for (I, VIII) and (I, IX);

buprenorphine — *P* < 0.05 for all pairs except for (I, II), (I, IV), (I, VI), (I, VII), (I, VIII), (I, IX) and (I, X);

other substance — *P* < 0.05 for all pairs except for (I, IV), (I, VI) and (I, IX);

### Social resource utilization and bystander involvement

We examined the extent of social resources used upon arrival at the EDs through the involvement of paramedics, police, security guards, child welfare or social workers (Table 3). Overall, paramedics participated in 27.9% of the total visits (95% CI: 26.9 – 28.9%) and police (including a few peace officers) were engaged in 5.1% of them (95% CI: 4.6 – 5.6%); security guards were involved in 2.3% of the visits (95% CI: 2.0 – 2.6%) and child welfare or social workers in 0.4% of them (95% CI: 0.3 – 0.6%). For the visits involving paramedics, police, or security guards, the proportion of non-prescribed opioid use without intention to cause harm tended to be higher than among the visits without each social resource. Compared to the visits without child welfare or social workers, the proportion of children’s exposure was higher among the visits involving child welfare or social workers.

We also examined if a visit was initiated by a bystander; meaning some patients were found on the street or in public places by a bystander (unknown to the patients) who sent the patients to ED or called police or paramedics. This accounted for 18.9% of the total visits (95% CI: 18.1 – 19.8%), the majority of which (61.4%) were associated with non-prescribed opioid use without intention to cause harm. Compared to the visits without bystander involvement, the proportion of non-prescribed opioid use without intention to cause harm was higher.

**Table 3:** Social resource utilization and bystander involvement

	Count (% of total visits) [95% CI]	Three most common opioid use contexts	
		**With resource**	**Without resource**
Paramedics	2,265 (27.9%) [26.9 – 28.9%]	1. 66.2% - Non-prescribed opioid use, without intention to cause harm 2. 20.3% - Unclear context, without intention to cause harm 3. 6.8% - Unclear context, undetermined intent to cause harm	1. 44.8% - Non-prescribed opioid use, without intention to cause harm 2. 21.5% - Unclear context, without intention to cause harm 3. 14.1% - Self-poisoning
Police (including peace officer)	413 (5.1%) [4.6 – 5.6%]	1. 58.6% - Non-prescribed opioid use, without intention to cause harm 2. 16.2% - Unclear context, without intention to cause harm 3. 11.4% - Self-poisoning	1. 50.4% - Non-prescribed opioid use, without intention to cause harm 2. 21.5% - Unclear context, without intention to cause harm 3. 11.5% - Self-poisoning
Security guard	183 (2.3%) [2.0 – 2.6%]	1. 65.0% - Non-prescribed opioid use, without intention to cause harm 2. 25.0% - Unclear context, without intention to cause harm 3. 8.2% - Unclear context, undetermined intent to cause harm	1. 50.5% - Non-prescribed opioid use, without intention to cause harm 2. 21.1% - Unclear context, without intention to cause harm 3. 11.7% - Self-poisoning
Child welfare or social worker	36 (0.4%) [0.3 – 0.6%]	1. 47.2% - Non-prescribed opioid use, without intention to cause harm 2. 19.4% - Children’s exposure 3. 11.1% - Self-poisoning	1. 50.8% - Non-prescribed opioid use, without intention to cause harm 2. 21.3% - Unclear context, without intention to cause harm 3. 11.5% - Self-poisoning
		***With bystander***	***Without bystander***
Bystander involvement	1,535 (18.9%) [18.1 – 19.8%]	1. 61.4% - Non-prescribed opioid use, without intention to cause harm 2. 26.8% - Unclear context, without intention to cause harm 3. 8.7% - Unclear context, undetermined intent to cause harm	1. 48.3% - Non-prescribed opioid use, without intention to cause harm 2. 19.9% - Unclear context, without intention to cause harm 3. 13.6% - Self-poisoning

Note: (1) For each social resource, the distribution of opioid use context was tested between the records with the social resource and those without: *P* < 0.05. (2) The distribution of opioid use context was tested between the records with bystander involvement and those without: *P* < 0.05

### Prior naloxone use

Naloxone use before arriving at EDs was mentioned in 1,896 visits (23.4%, 95% CI: 22.5 – 24.3%) (Table 4), the majority of which (54.3%) were associated with non-prescribed opioid use without intention to cause harm. Among all visits with this opioid use context, the proportion of prior naloxone use was 25.0%. The highest proportion of prior naloxone use (41.1%) was seen among the visits associated with unclear opioid use context without intention to cause harm.

Naloxone was administered by a variety of persons. Table 4 shows the types of naloxone administrators, such as health care workers, family members, friends, acquaintance, shelter/group home staff, public place staff, law enforcement staff, bystanders or even patients themselves. In some situations, multiple persons or even multiple types of persons were involved. When the narrative mentioned multiple types, but did not specify who administered naloxone, we classified the type as “Other”. In this situation, the majority of records (90.5%) mentioned emergency medical service. When no information could be used for classification, the type was “Unknown”, which comprised 35.4% (*n* = 671) of the records with prior naloxone use. Among those records where we identified administrators (*n* = 1,225), health care workers (only) constituted 35.5% (435 of 1,225) while bystanders (only) represented 15.8% (194 of 1,225) of the naloxone administrators.

**Table 4:** Prior naloxone use and administrator type

Administrator type		Opioid use context								
		All	I. Non-prescribed opioid use, without intention to cause harm	VII. Unclear context, without intention to cause harm	X. Unclear context, undetermined intent to cause harm	VIII. Self-poisoning	III. Children’s exposure	II. Medication error	IX. Poisoned by others	VI. Other context, without intention to cause harm
	*n* ^#^	1896	1029	707	102	32	14	9	2	1
	Distribution of opioid use context	100%	54.3%	37.3%	5.4%	1.7%	0.7%	0.5%	0.1%	0.1%
	% among specific opioid use context [95% CI]	23.4% [22.5 – 24.3%]	25.0% [23.7 – 26.3%]	41.1% [38.8 – 43.4%]	14.2% [11.8 – 17.0%]	3.4% [2.4 – 4.8%]	4.7% [2.8 – 7.9%]	4.2% [2.3 – 7.9%]	11.1% [2.4 – 38.9%]	3.1% [0.4 – 20.9%]
		n (column %)	n (column %)	n (column %)	n (column %)	n (column %)	n (column %)	n (column %)	n (column %)	n (column %)
1. Health care workers^a^		435 (22.9%)	298 (29.0%)	99 (14.0%)	19 (18.6%)	8 (25.0%)	4 (28.6%)	7 (77.8%)	0 (0%)	0 (0%)
2. Family members, friends, or acquaintance		176 (9.3%)	119 (11.6%)	43 (6.1%)	6 (5.9%)	4 (12.5%)	2 (14.3%)	1 (11.1%)	1 (50%)	0 (0%)
3. Shelter/group home staff		159 (8.4%)	113 (11.0%)	31 (4.4%)	13 (12.7%)	2 (6.3%)	0 (0%)	0 (0%)	0 (0%)	0 (0%)
4. Public place staff^b^		18 (0.9%)	12 (1.2%)	5 (0.7%)	1 (1.0%)	0 (0%)	0 (0%)	0 (0%)	0 (0%)	0 (0%)
5. Law enforcement staff^c^		105 (5.5%)	57 (5.5%)	40 (5.7%)	6 (5.9%)	1 (3.1%)	0 (0%)	0 (0%)	0 (0%)	1 (100%)
6. Self		9 (0.5%)	6 (0.6%)	0 (0%)	1 (1.0%)	2 (6.3%)	0 (0%)	0 (0%)	0 (0%)	0 (0%)
7. Bystanders^d^		194 (10.2%)	120 (11.7%)	53 (7.5%)	19 (18.6%)	2 (6.3%)	0 (0%)	0 (0%)	0 (0%)	0 (0%)
8. Multiple^e^		24 (1.3%)	16 (1.6%)	5 (0.7%)	2 (2.0%)	1 (3.1%)	0 (0%)	0 (0%)	0 (0%)	0 (0%)
9. Other^f^		105 (5.5%)	65 (6.3%)	31 (4.4%)	5 (4.9%)	1 (3.1%)	3 (21.4%)	0 (0%)	0 (0%)	0 (0%)
10. Unknown		671 (35.4%)	223 (21.7%)	400 (56.6%)	30 (29.4%)	11 (34.4%)	5 (35.7%)	1 (11.1%)	1 (50%)	0 (0%)

Note:

^a^ This type includes health care workers, such as doctors, nurses, staff from paramedics (majorly emergency medical service and few firefighters), safe injection sites, mental health service or personal care service.

^b^ This type includes staff working in restaurants, retail stores, hotels, public transportation systems, and community centres.

^c^ This type includes police officers, peace officers, community safety officers, and security guards.

^d^ This type includes persons who were unknown to patients and not in any of Type 1, 3, 4, 5.

^e^ This type includes where two or more of Type 1–7 were involved.

^f^ This type includes where anyone in Type 1–7 administered naloxone but the exact type was unknown. The majority (90.5%) involved emergency medical service or others.

^#^ Among the patients where naloxone use was mentioned, naloxone had an effect on 61.7% of them, no effect on 2.2% of them. The effect could not be discerned among 36.1% of the patients.

The distribution of administrator type was tested between Category I (reference) and each of the other categories: *P* > 0.05 for all pairs except for (I, VII).

### Distribution of ED presentations by opioid use context

Table 5 shows the distribution of ED presentations (poisoning effects, withdrawal symptoms, and co-occurring injuries) by opioid use context. A poisoning effect means toxicity caused by overdose of a substance. Since the ED visits we examined may also involve other substances, poisoning effects were not limited to those of opioid overdose such as pinpoint pupils, respiratory depression, and a decreased level of consciousness.

The majority of patients (92.7%, 95% CI: 92.1 – 93.3%) presented with poisoning effects, either with poisoning effects only or with injuries. The distribution of ED presentations was different among opioid use contexts. For example, the proportion of poisoning effect combined with injuries was higher among the visits with non-prescribed opioid use without intention to cause harm than the visits with medication error or self-poisoning.

1% of patients (95% CI: 0.9 – 1.3%) did not present with poisoning effects, but with withdrawal symptoms. 5% of patients (95% CI: 4.8 – 5.8%) came to the ED with injuries related to opioid use without an opioid poisoning effect. Among a few patients (0.7%, 95% CI: 0.5 – 0.9%), the main purpose of the ED visit was related to opioids, but there were no poisonings or injuries related to opioids. For example, they came to request rehabilitation, to refill an opioid prescription, or for screening opioids.

For those who presented with both poisoning effects and injuries, the injuries could be related to opioid use or not. The situations included either an unintentional or self-inflicted injury under the influence of opioids, or an injury sustained during a conflict or sexual assault related to opioids, or an injury sustained during the opioid administration process (such as cuts, burns, etc.), or an injury not related to opioids at all.

**Table 5:** Distribution of ED presentations by opioid use context

**A. Presenting poisoning effect** (*n*** = 7,522 % of total visits [95% CI] = 92.7% [92.1 – 93.3%] )**				
opioid use context	**A1**. poisoning effect only	**A2**. with (1) unintentional or self-inflicted injuries under the influence of opioids; or (2) injuries sustained during a conflict related to opioids; or (3) being sexually assaulted related to opioids	**A3**. with injuries (cuts, burns etc.) sustained during opioid administration	**A4**. with injuries not related to opioid use
*I. Non-prescribed opioid use*,* without intention to cause harm*	3,296 (88.2%)	341 (9.1%)	5 (0.1%)	97 (2.6%)
*II. Medication error*	170 (94.4%)	10 (5.5%)	0 (0%)	2 (1.1%)
*III. Children’s exposure*	278 (100%)	0 (0%)	0 (0%)	0 (0%)
*IV. Mother’s opioid use*	10 (100%)	0 (0%)	0 (0%)	0 (0%)
*V. Medical treatment*	13 (81.3%)	3 (18.7%)	0 (0%)	0 (0%)
*VI. Other context*,* without intention to cause harm*	30 (100%)	0 (0%)	0 (0%)	0 (0%)
*VII. Unclear context*,* without intention to cause harm*	1,580 (97.8%)	18 (1.1%)	9 (0.6%)	8 (0.5%)
*VIII. Self-poisoning*	911 (99.0%)	9 (1.0%)	0 (0%)	0 (0%)
*IX. Poisoned by opioids*	12 (66.7%)	6 (33.3%)	0 (0%)	0 (0%)
*X. Unclear context*,* undetermined intent to cause harm*	711 (99.6%)	2 (0.3%)	0 (0%)	1 (0.1%)
**B. Presenting opioid withdrawal effect** (*n* = 89 % of total visits [95% CI] = 1.1% [0.9 – 1.3%])				
opioid use context	**B1.** withdrawal effect only	**B2.** with unintentional or self-inflicted injuries caused by withdrawal effect	**B3.** with injuries (cuts, burns etc.) sustained during opioid administration	**B4.** with injuries not related to opioid
*I. Non-prescribed opioid use*,* without intention to cause harm*	54 (96.4%)	1 (1.8%)	0 (0%)	1 (1.8%)
*V. Medical treatment*	18 (90%)	1 (5%)	0 (0%)	1 (5%)
*VII. Unclear context*,* without intention to cause harm*	11 (84.6%)	1 (7.7%)	0 (0%)	1 (7.7%)
**C. no opioid poisoning effect presented**,** but the injuries were related to opioid use** (*n* = 427 % of total visits [95% CI] = 5.3% [4.8 – 5.8%])				
opioid use context		**C1.** (1) unintentional or self-inflicted injuries under the influence of opioids; or (2) injuries sustained during a conflict related to opioids; or (3) being sexually assaulted related to opioids	**C2.** Needle-related skin problems or burns sustained during opioid administration	
*I. Non-prescribed opioid use*,* without intention to cause harm*		254 (87.9%)	35 (12.1%)	
*II. Medication error*		33 (100%)	0 (0%)	
*V. Medical treatment*		8 (100%)	0 (0%)	
*VI. Other context*,* without intention to cause harm*		2 (100%)	0 (0%)	
*VII. Unclear context*,* without intention to cause harm*		87 (98.9%)	1 (1.1%)	
*VIII. Self-poisoning*		5 (100%)	0 (0%)	
*X. Unclear context*,* undetermined intent to cause harm*		2 (100%)	0 (0%)	
**D. The main purpose of ED visit was related to opioids (e.g. to request rehabilitation**,** to refill opioid prescription**,** for screening)**,** but there were no poisonings or injuries related to opioids. The patient could have injuries not related to opioids.** (*n* = 56 % of total visits [95% CI] = 0.7% [0.5 – 0.9%])				
opioid use context		**D1.** no poisonings/injuries	**D2.** with injuries not related to opioid use	
*I. Non-prescribed opioid use*,* without intention to cause harm*		17 (85%)	3 (15%)	
*III. Children’s exposure*		16 (94.1%)	1 (5.9%)	
*V. Medical treatment*		7 (77.8%)	2 (22.2%)	
*VII. Unclear context*,* without intention to cause harm*		3 (100%)	0 (0%)	
*VIII. Self-poisoning*		2 (40%)	3 (60%)	
*X. Unclear context*,* undetermined intent to cause harm*		1 (50%)	1 (50%)	

Note:

1. The percentage in bracket in each cell is the row percentage.

2. The nature of injury information is missing for 18 cases that are excluded from the Table.

3. For 11 visits, the main purpose was to request rehabilitation; for another 23 visits, rehabilitation was requested other than treatment.

4. For each opioid use context in Section A, the distribution of ED presentations was tested between Category I (reference) and each of the other categories: *P* < 0.05 for all pairs except for (I, IV) and (I, V).

5. For each opioid use context in Section B, the distribution of ED presentations was tested between Category I (reference) and each of the other categories: *P* > 0.05 for all pairs.

6. For each opioid use context in Section C, the distribution of ED presentations was tested between Category I (reference) and each of the other categories: *P* < 0.05 for (I, II) and (I, VII), *P* > 0.05 for all the other pairs .

7. For each opioid use context in Section D, the distribution of ED presentations was tested between Category I (reference) and each of the other categories: *P* > 0.05 for all pairs.

## Discussion

This study used a mixed methods design to study the contextual factors and co-occurrence of poisonings and injuries among the opioid-related ED visits from 2011 to 2022 from a national injury and poisoning sentinel surveillance system in Canada. The most common opioid use context was non-prescribed opioid use without intention to cause harm, followed by self-poisoning, children’s exposure, and medication error. Paramedics participated in 27.9% of cases. Police and security guards were involved in 5.1% and 2.3% of cases, respectively. Child welfare or social workers were involved in 0.4% of cases. Among 18.9% of cases, the visit was initiated by a bystander. Naloxone use before arriving at the ED occurred in 23.4% of cases and a variety of persons administered the naloxone. The majority of patients presented with poisoning effects, either with poisoning effects only or in conjunction with injuries or other conditions.

To our knowledge, no published studies have probed the in-depth contexts surrounding poisoning and injury-related ED visits associated with opioids in Canada. The National Ambulatory Care and Reporting System (NACRS) [28], an administrative database, was often used in Canadian ED visit studies, but it did not have the same level of detail as CHIRPP. NACRS has very limited data elements describing what happened before the ED visits. CHIRPP has variables detailing injury and poisoning events and a free-flow narrative that allows recording multiple facets of the visits, so we were able to use it to examine the contextual factors surrounding poisoning and injury-related ED visits associated with opioids. We have identified some opioid use contexts that were rarely described in other studies. We have also examined the extent of social resource utilization, bystander involvement, and prior naloxone use which were hardly reported before.

Identifying social resource involvement has provided a picture of social burden, other than health care burden, of these visits. As high as 18.9% of the ED visits initiated by a bystander shows the high risk of the population suffering from opioid-related poisonings and injuries, which highlights the recommendation by the Canadian Association of Emergency Physicians that ED care providers play a role in identifying patients with social factors that may inhibit engagement in treatment [16]. 

Our study also provides a basic understanding of the magnitude of prior naloxone use and types of administrators among opioid-related ED visits. Naloxone is vital in reversing opioid overdose for saving lives. In Canada, naloxone is used by health care providers in the healthcare settings. Take-home naloxone kits are also available to anyone who may be at risk of an overdose or who is likely to encounter one. Take-home naloxone kits are available without a prescription and can be picked up at most pharmacies or local health authorities [29]. Our data show that naloxone was administered to patients before arriving at EDs among 23.4% of poisoning and injury-related ED visits associated with opioids. We consider several factors when interpreting this number. First, recording naloxone use information is not mandatory for CHIRPP, so we consider it as “at least 23.4%”. Second, not all the persons who experienced overdose that was reversed by naloxone came to EDs, so this number is not the proportion of naloxone use among community overdose cases. People who use *drugs* comprise a substantial portion of community overdose responders [30]. Even though the Canadian guidance [30] recommends that responders call emergency medical service even when an overdose is reversed, many people who use drugs do not feel safe calling emergency medical service [30]. We expect many overdose cases did not receive ED care. Our data show that a variety of persons other than health care workers administered naloxone during overdose events, such as family members, friends, acquaintance, shelter/group home staff, law enforcement staff, public place staff, bystanders, and patients themselves. Of note, bystanders comprised a considerable portion of the naloxone administrators. This shows that take-home naloxone kits are widely used and playing an important role in saving lives in Canada.

ED presentations among opioid-related visits can be complex, as shown in our study. Other than poisoning effects, some patients presented with injuries that were either associated with opioid use or not. Those injuries associated with opioid use were caused by the opioid administration process (such as burns, cuts, etc.) or could be linked to impairment on neuropsychological executive function (such as fall, motor vehicle crash, struck by etc.) [31]. Some patients also presented with injuries related to opioid use without poisoning effects. The complexity of scenarios requires ED health care practitioners to perform a comprehensive assessment of a patient’s conditions in order to address their needs. Our study shows that some patients came to the EDs to request rehabilitation. The collaboration of EDs with community-based providers, addiction clinics, and local supports is important for individuals with opioid use disorder [16, 17, 19, 32]. 

This paper was intended to be the first in a series to provide the contextual factors surrounding the poisoning and injury-related ED visits associated with opioids, depicting an overall picture from 2011 to 2022. For some categories and sub-categories of opioid use context, we will dive deeper into the characteristics. For example, we have found that some patients with opioid agonist therapy used methadone/suboxone concomitantly with other *drugs*. We will examine the characteristics of those patients, methadone/suboxone providers, duration of use, types of concomitant drugs, reasons for using other drugs, and care barriers, etc. The information would be useful for making policies regarding opioid agonist therapy. Also, this study used the overall data from 2011 to 2022. It would be helpful to study specific time periods to examine the changes over time in the future.

### Limitations

There are several limitations in this study. First, because CHIRPP is a sentinel surveillance system, generalizability is restricted; rural and Indigenous populations are under-represented in CHIRPP [20, 33]. Second, due to CHIRPP’s reporting being voluntary, socioeconomic and resource access bias for substance users may play a role in this study, such as stigma and healthcare access. Stigma about substances and substance users may cause those experiencing difficulties with their usage to avoid going to EDs. Stigma might also affect reporting behaviour [34, 35]. All of these might cause an underreporting. Third, pediatric hospitals accounted for 55% of the CHIRPP reporting-hospitals [21], so the opioid use contexts (such as non-prescribed opioid use without intention to cause harm) that most likely occurred in the adult population were under-represented in the study. Fourth, since CHIRPP is an injury and poisoning surveillance system, opioid use disorder-related ED visits and co-morbidities other than poisonings and injuries were not captured in CHIRPP. Fifth, our data about social resource and prior naloxone use are an underestimation since reporting those in CHIRPP is not mandatory. Lastly, data processing delays can affect the data completeness, majorly for 2022.

## Conclusions

We used a mixed methods study to examine the contextual factors and co-occurrence of poisonings and injuries among the opioid-related ED visits from 2011 to 2022 from a national injury and poisoning sentinel surveillance system in Canada. We have identified some rare opioid use context, provided information about social resource utilization, bystander involvement, and prior naloxone use, and shown the complexity of ED presentations. The evidence from this study can inform ED programming and opioid-related poisoning and injury intervention and prevention. It can also guide further research on the opioid-related ED visits.

## Electronic supplementary material

Below is the link to the electronic supplementary material.

Supplementary Material 1

Supplementary Material 2

## Data Availability

The datasets used and analyzed during the current study are available from the corresponding author on reasonable request with privacy and confidentiality respected.

## Abbreviations

CHIRPP: Canadian hospitals injury reporting and prevention program
ED: Emergency department
CI: Confidence interval
NACRS: National ambulatory care and reporting system
