# Supplementary material for: A mixed methods study on poisoning and injury-related emergency department visits associated with opioids in Canada, 2011 to 2022: from the Canadian hospitals injury reporting and prevention program
Source: BMC Public Health. 2024 Sep 18;24:2546. doi: 10.1186/s12889-024-20016-8 (PMC11411743; doi:10.1186/s12889-024-20016-8)
Supplement: Supplementary file 1 — Supplementary Material 1 [file 12889_2024_20016_MOESM1_ESM.docx]

**Appendix 2**

**Inter-rater reliability test**

The following table presents the statistics of inter-rater reliability test. We first tested the manual review process for identifying poisoning/injury-related ED visits associated with opioids (n=1,327). Then, we tested 1101 cases that were identified by both raters (AR and JM) as opioid-related cases. For some cases, the opioid use context was discussed and determined through a consensus. We have deleted those cases (n=67) for testing opioid use context.

|  | sample size | Gwet's AC_1_ | | | Cohen's Kappa | | |
| --- | --- | --- | --- | --- | --- | --- | --- |
|  |  | AC_1_ | 95% confidence interval | | Kappa | 95% confidence interval | |
| Manual review to identify opioid-related ED visits | 1327 | 0.97 | 0.96 | 0.98 | 0.91 | 0.88 | 0.94 |
| Opioid use context | 1034 | 0.92 | 0.90 | 0.94 | 0.77 | 0.72 | 0.82 |
| Paramedic involvement | 1101 | 0.93 | 0.91 | 0.95 | 0.93 | 0.91 | 0.95 |
| Police involvement | 1101 | 0.98 | 0.98 | 0.99 | 0.85 | 0.77 | 0.92 |
| Security guard involvement | 1101 | 0.99 | 0.99 | 1.00 | 0.93 | 0.88 | 0.98 |
| Child welfare/social worker involvement | 1101 | 1.00 | 1.00 | 1.00 | 0* | 0* | 0* |
| Bystander involvement | 1101 | 0.69 | 0.65 | 0.73 | 0.52 | 0.47 | 0.57 |
| Naloxone use | 1101 | 0.96 | 0.94 | 0.98 | 0.96 | 0.94 | 0.97 |

*The value shows the Kappa paradox when a prevalence is close to 0% or 100%. Gwet’s AC_1_ is a more appropriate statistics for testing the reliability.

**Reference:**

Gwet, K. L. (2008). Computing inter‐rater reliability and its variance in the presence of high agreement. *British Journal of Mathematical and Statistical Psychology*, 61(1). https://doi.org/10.1348/000711006x126600

Gwet, K. L. (2021). *Handbook of inter-rater reliability* (5th ed.). Gaithersburg, MD: AgreeStat Analytics.
