# Supplementary material for: A mixed methods study on poisoning and injury-related emergency department visits associated with opioids in Canada, 2011 to 2022: from the Canadian hospitals injury reporting and prevention program
Source: BMC Public Health. 2024 Sep 18;24:2546. doi: 10.1186/s12889-024-20016-8 (PMC11411743; doi:10.1186/s12889-024-20016-8)
Supplement: Supplementary file 2 — Supplementary Material 2 [file 12889_2024_20016_MOESM2_ESM.docx]

**Appendix 1**

**SAS search terms for identifying opioid-related records**

pattern=prxparse('/

\s222[\s\.]|

\s282[\s\.]|

\s292[\s\.]|

[^t]ox[iy][ck]o|

[ck]odal|

[ée]torphine|

\sm\.?\s?o\.?\s?s\.?(\s|-sr)\s|

\sw(\s|\/|ith\s)cod|

a\.?\s?c\.?\s?(&|and)\s?c\.?\s|

ac[eé]morph|

ac[eé]t\s?[23]|

Actacode|

Airacof|

atasol|

avinza|

Axacet|

Axisal|

azocine|

b[eé]zitramide|

bion[io]ne|

Bisoltus|

bolodorm|

bremazocine|

Bromophar|

brompton|

Bronchicum|

Bronchodine|

bunavail|

buphine|

bupr[eé]norphine|

butorphanol|

c\.?\s?(&|and)\s?C\.?\s|

calmylin|

CapCof|

cardanon|

co-?codamol|

co[nd]orfone|

co\s?-?dydramol|

coactifed|

Cocet|

cod[eé]?in|

codal|

Codant|

Codar|

Codedrill|

Codeisan|

codenon|

Codeprex|

Coderpina|

codi[eé]ne|

Codicalm|

Codicept|

Codinex|

codix\s?5|

codone|

Codrix|

Coducept|

contin\s|

Cotabs|

cotridin|

Cotrifed|

Cougel|

\sCoutan\s|

Covan Syrup|

cyclazocine|

demerol|

dextromoramide|

dextrorphane?|

Dex-Tuss|

dezocine|

diacephine|

diaphorin|

diconal|

dihydro|

dim[eé]thylthiambutene|

Dimetapp|

dinarkon|

Dinco|

diph[eé]noxylate|

dolonovag|

dolophine|

drocode|

durela|

Empracet|

emtec|

enadoline|

EndaCof|

Endal|

endocet|

endodan|

endone|

enk[ée]phaline?|

ethoheptazine|

etonitazene|

etoxeridine|

eubine|

euk?din|

eurodamine|

eutagen|

exalgo|

Exdol|

ExeClear|

Expecto|

Expectorant|

Farmacod|

faxeladol|

fenta|phenta|

fentora|

Fiorinal[\s-]C|

fur[eé]thidine|

Galcodine|

Ganituss|

gelonida|

gesic|

Guaifenesin|

h[eé]ro[iï]n|

Histex|

hydromo|

hydrocodone|

hydrocont|

hydrostat|

hysingla|

instanyl|

ionsys|

Iophen|

isalmadol|

isonip[eé]caine|

isotonitazene|

jurnista|

k[eé]tazocine|

k[eé]tob[eé]midone|

kadian |

ketogan|

kyotorphine?|

l[eé]f[eé]tamine|

l[eé]vorphanol|

lau?did|

lauda(co)?num|

lenoltec|

leptanal|

Lexuss|

lop[eé]ramide|

lorcet|

lortab|

Lortuss|

ludonal|

m-?eslon |

m[eé]p[eé]ridine|

m[eé]ridine|

m[eé]tazocine|

m[eé]thado|

m[eé]thadyle?|

m[eé]thylsamidorphan|

Mar[\s-]cof|

Maxi[\s-]Tuss|

M-clear|

medicodal|

M-end|

meptazinol|

Mesehist|

metadol|

Methoxacet|

Methoxisal|

mor(ph|f)|

\sms contin\s|

ms[\s-\.]ir\s|

muscle\s?(&|and)\sback\spain\srel|

naloxo|

narcan|

narcobasin[ae]|

nargenol|

narodal|

Nasotuss|

Neo AC|

Ninjacof|

norco|

norphine|

Notuss|

Novahistex|

novolaudon|

nsc\s?19043|

Nu[\s-]acetaco|

nucodan|

nucynta|

olic[eé]ridine|

omnopon|

onsolis|

opiate|opioid|opium|

opton|

Oradrine|

oripavine|

ossicodone|

oxanest|

oxecta|

\soxy\s|

\soxyco|

\soxy[\s\.]?ir|

\soxydose\s|

\soxyfast\s|

\soxygesic\s|

\soxykon\s|

\soxymorph\s|

\soxyneo\s|

\soxynorm\s|

p[eé]thidine|

Pain Relief|

Painex|

pal[il]adon|

pancodine|

papaver|pavot|

Parafon|

paregoric|

pavinal|

pecfent|

Pectoral|

pentazocine|

Pentuss|

perc[ao]cet|

percodan|

percolone|

ph[eé]nadoxone|

ph[eé]nazocine|

ph[eé]ncyclidine|

Phenaphen|

phenaridine|

Phenylhistine|

Phrenilin|

pic[eé]nadol|

piminodine|

pipa[dn]one|

Pluratuss|

PMS[\s-]pharnal|

Poly Hist|

Pr[ao]cet|

Pro[\s-]clear|

Pro[\s-]red|

profadol|

promedol|

Pronal|

pronarcin|

propiram|

propoxyph[eè]ne|

Pseudodine|

ralivia|

ramadol|

rapinyl|

recuvyra|

Relcof|

remoxy|

Robafen|

Robaxacet|

Robaxisal|

Robitussin|

Rounox|

Routec|

roxicet|

Rydex|

samidorphan|

sedapain|

semcox|

sinthiodal|

Sinutab|

\sSoma\s|

sophidone|

Spasmhalt|

statex|

Statuss|

stupenal|

suboxone|

subutex|

supeudol|

talwin|

Tamicode|

tanyl|

tapentadol|

targin|

tebodal|

tecnal|

tekodin|

Terpin|

Teva[\s-]cotridin|

th[eé]ba[iï]ne|

thecodin|

tifluadom|

tilidine|

Tl[\s-]hist|

tonazocine|

tramacet|

tramadol|

tramide|

Triacin|

Trianal|

Triatec|

Tricode|

tridural|

Tusnel|

Tussaminic|

tussin|

Tussoret|

Tuxarin|

Tuzistra|

tylenol\s?#\s?[1-4]\s|

tylenol\s?[1-4]\s[^(tablet)]|

tylenol\sno\.?\s?[1-4]\s|

tylenol\sw(\s|\/|ith\s)cod|

U-47700|

ultram|

vic[ao]din|

Wampole|

wildnil|

Z[\s-]tuss|

Zodryl|

zohydro|

Zotex|

zubsolv|

zytram|

etazene|

brorphine|

metodesnitazene|

nortilidine|

metonitazene|

carbonyl[\s-]bromadol|

AP[\s-]238|

O[\s-]AMKD|

isotonitazene|

2[\s-]methyl[\s-]AP[\s-]237|

tianeptine|

piperidylthiambutene|

protonitazene|

2F[\s-]viminol|

vopac

/i');
